# Multivalent Engagement of TFIID to Nucleosomes

**DOI:** 10.1371/journal.pone.0073495

**Published:** 2013-09-11

**Authors:** Rick van Nuland, Andrea W. Schram, Frederik M. A. van Schaik, Pascal W. T. C. Jansen, Michiel Vermeulen, H. T. Marc Timmers

**Affiliations:** 1 Department of Molecular Cancer Research, University Medical Center Utrecht, Utrecht, The Netherlands; 2 Netherlands Proteomics Center, Utrecht, The Netherlands; 3 Department of Medical Oncology, University Medical Center Utrecht, Utrecht, The Netherlands; Université Paris-Diderot, France

## Abstract

The process of eukaryotic transcription initiation involves the assembly of basal transcription factor complexes on the gene promoter. The recruitment of TFIID is an early and important step in this process. Gene promoters contain distinct DNA sequence elements and are marked by the presence of post-translationally modified nucleosomes. The contributions of these individual features for TFIID recruitment remain to be elucidated. Here, we use immobilized reconstituted promoter nucleosomes, conventional biochemistry and quantitative mass spectrometry to investigate the influence of distinct histone modifications and functional DNA-elements on the binding of TFIID. Our data reveal synergistic effects of H3K4me3, H3K14ac and a TATA box sequence on TFIID binding *in vitro*. Stoichiometry analyses of affinity purified human TFIID identified the presence of a stable dimeric core. Several peripheral TAFs, including those interacting with distinct promoter features, are substoichiometric yet present in substantial amounts. Finally, we find that the TAF3 subunit of TFIID binds to poised promoters in an H3K4me3-dependent manner. Moreover, the PHD-finger of TAF3 is important for rapid induction of target genes. Thus, fine-tuning of TFIID engagement on promoters is driven by synergistic contacts with both DNA-elements and histone modifications, eventually resulting in a high affinity interaction and activation of transcription.

## Background

RNA polymerase II (pol II) mediates the transcription of all protein coding genes in eukaryotic cells. Activation of transcription by sequence-specific DNA-binding transcription factors leads to recruitment of basal transcription factors to core promoters that together establish the pre-initiation complex (PIC) [1]. PIC assembly is initiated by core promoter association of the TFIID complex, followed by the sequential binding of other basal factors and recruitment of pol II [2]. TFIID is a large complex and contains ∼13 TBP associated factors (TAFs) and the TATA binding protein (TBP) [3]. 9 of the 13 TAFs contain a histone fold dimerization domain that allows multiple pairwise interactions within the complex [4], [5]. TFIID adopts a clamp-like shape that features a symmetrical core. The TFIID complex has been studied extensively in yeast using multistep affinity purified complexes. Coomassie staining based analysis of these complexes revealed that a subset of TAFs (TAF4, TAF5, TAF6, TAF9, TAF10, TAF11 and TAF12) are present in more than one copy [6]. Recent work on reconstituted human TFIID confirmed these results and showed that upon addition of the TAF8/TAF10 dimer, a new surface is created that allows the assembly of single copies of the other TAFs to form a full TFIID complex [7]. Structural heterogeneity has been observed in TFIID preparations isolated from human cells and this was linked to a sub-stoichiometric TAF2 presence [8]. Additionally, the binding of TFIID to DNA induces a structural rearrangement within the complex [9]. The mechanism for this remains to be elucidated and it might be influenced by changes in TFIID composition.

Several subunits within TFIID can bind to specific DNA-elements found at promoters. TBP interacts with the TATA element, which is found upstream of the transcription start site (TSS). In yeast as well as in mammals, only a subset of genes contains a high affinity TATA box sequence [10], [11]. Surprisingly, in yeast, TFIID association with promoters is inversely correlated with the presence of a consensus TATA sequence [12]. TBP association with TFIID and the TATA sequence is stabilized by binding of the TFIIA complex [13], [14]. Additionally, TAFs1/2 interact with the initiator element (INR) [15] and TAFs6/9 can bind to a downstream promoter element (DPE) [16].

Chromatin has an important role in the regulation of transcription. The basic building block of chromatin is the nucleosome, comprised of an octamer of histone proteins. Posttranslational modifications on the protruding tails of histones contribute to transcription regulation. Effector proteins that contain specific binding modules can recognize these chemical modifications and get recruited to genomic loci [17]–[19]. Tri-methylation of lysine 4 on histone H3 (H3K4me3) is associated with virtually all active and poised promoters both in yeast and in mammals [20], [21]. Several H3K4me3 binding proteins have been identified, including the chromatin remodeler BPTF and the TFIID subunit TAF3. Binding of these proteins to H3K4me3 occurs through their plant homeodomain (PHD) fingers [22]–[24]. Recently, it has been described that the TAF3-H3K4me3 interaction in mammals is required for PIC assembly on a selective group of genes which are mainly involved in the response to DNA damage [25]. In addition to H3K4me3, promoter-associated modifications include hyperacetylation on histone H3 and the presence of a specific histone variant H2A.Z, which replaces the canonical H2A [20], [26].

Here we show that the binding of TFIID to recombinant nucleosomes is synergistically enhanced by the presence of a TATA box in nucleosomes carrying H3K_C_4me3 and H3K14ac. However, this binding is not affected by incorporation of histone variant H2A.Z or the H3K27me3 repressive mark. To further dissect the biochemistry of TFIID and to investigate the requirements for TFIID binding to nucleosomes *in vitro*, we determined the stoichiometry of endogenous human TFIID. These experiments revealed that TFIID consists of a stable symmetric core and a number of peripheral sub-stoichiometric TAFs. Finally, we show that binding of TAF3 is enriched on ‘poised’ stress gene promoters containing H3K4me3 in a PHD-finger dependent manner *in vivo*.

## Results

### TFIID binding to H3K4_C_me3 nucleosomes

**Figure 1:**
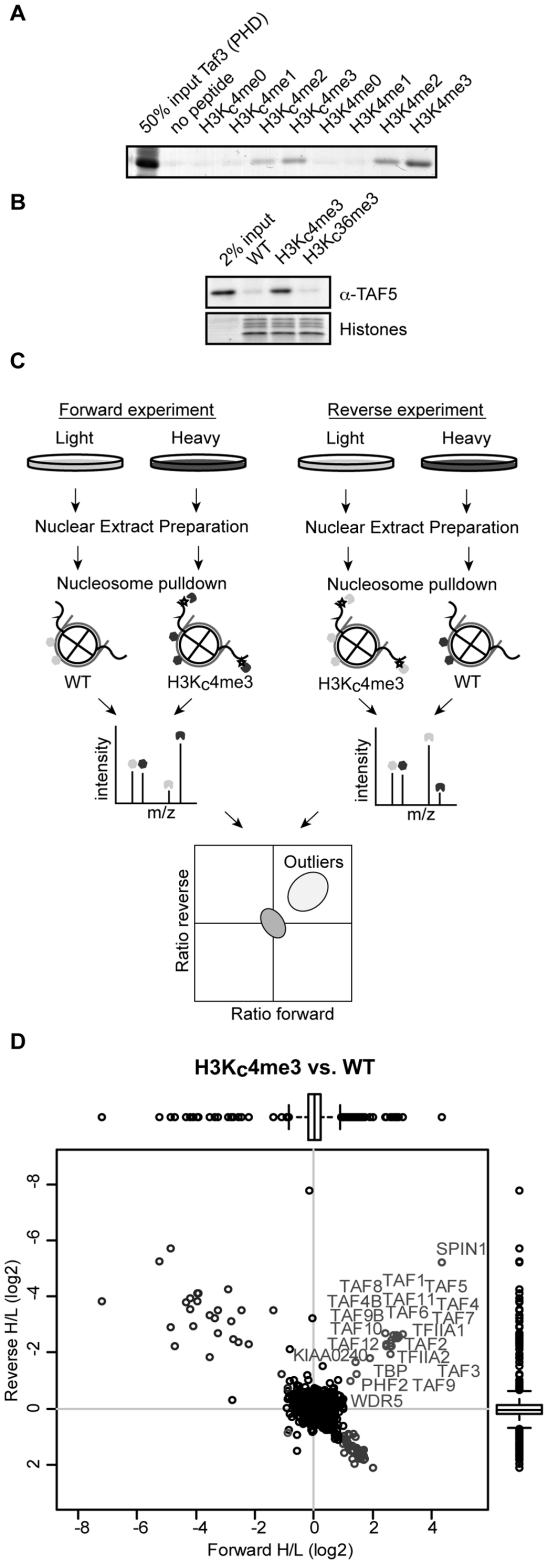
H3K4_C_me3 nucleosomes bind endogenous TFIID and recombinant TAF3. (A) Pulldown with the indicated biotinylated peptides using streptavidin coated beads incubated with GST-TAF3 PHD protein lysates. Proteins are visualized using Coomassie blue staining. (B) Immunoblot analysis of endogenous TAF5 binding to immobilized recombinant nucleosomes with the indicated MLA modification. Histones are visualized using Coomassie blue staining. (C) Workflow as applied for quantitative analysis of nucleosome interactors. In brief, heavy and light labeled extracts are used for pull-downs with immobilized, differentially modified nucleosomes. Experiments are also performed with a label swap. Eluted proteins are measured using LC-MS/MS. Enriched proteins in both experiments are selected based on box plot statistics. (D) Scatter plot of SILAC ratios for H3K4_C_me3 versus non-modified nucleosome interacting proteins. Upper right corner significant outliers are depicted and labeled based on the box plot analysis.

TFIID is a large protein complex containing various subunits that can interact with specific DNA-elements and distinct histone modifications. Thus far, such interactions have mainly been studied using gel-shift and peptide pull-down assays [1], [16], [23], [27]. Recently, several approaches have been developed to generate *in vitro* reconstituted nucleosomes containing specific histone modifications and DNA sequences [28], [29]. In combination with quantitative mass spectrometry, affinity purifications using such immobilized nucleosomes can reveal proteins and protein complexes that can specifically interact with these *in vitro* assembled modified nucleosomes species [6], [30], [31]. We applied a methyl lysine analog (MLA) approach to produce recombinant nucleosomes carrying an H3K4me3 mimic (H3K_C_4me3) with the aim to use these as bait for affinity purifications in crude nuclear extracts. To validate our approach we first tested the interaction between the TAF3 PHD-finger and different MLA peptides. As shown in Fig. 1A, the TAF3 PHD-finger specifically binds to the histone H3 N-terminus containing the H3K_C_4me2 and H3K_C_4me3 modification analogs. This binding is specific and comparable to H3 peptides containing natural methylated lysines (H3K4me2 and H3K4me3). This indicates that the MLA approach can be used as a tool to study TFIID-nucleosome interactions.

Next, we reconstituted MLA containing histone octamers with the ‘Widom’ 601 sequence labeled with a biotin on the 5′-end. The ‘Widom’ 601 sequence was used to prevent unintentional sliding of the nucleosome and transcription factor binding. Furthermore, the ‘Widom’ 601 sequence allows for efficient reconstitution of nucleosomes. Reconstituted nucleosomes were immobilized on streptavidin-conjugated magnetic beads and incubated with HeLa nuclear extract. To validate our assay we used western blotting to show the specific binding of the TFIID core subunit TAF5 to H3K_C_4me3 containing nucleosomes. In contrast, TAF5 does not interact with unmodified or H3K36_C_me3 marked nucleosomes, which validates the specificity of our approach (Fig. 1B).

The H3K_C_4me3 and unmodified control nucleosomes were then used for affinity purification in combination with SILAC-labeled HeLa nuclear extracts. Quantitative mass spectrometry was applied to identify specific interactors in an unbiased manner [32] (Fig. 1C). Nucleosomes with H3K4_C_me3 showed enriched binding of all TFIID subunits and TBP (Fig. 1D). The SILAC ratio plots also reveal specific binding of TFIIA, which is known to functionally cooperate with TFIID during the early stages of PIC assembly. Several known H3K4me3 interactors were also identified, including PHF2 and SPIN1 [30], [32]. In contrast, a number of known H3K4me3 interactors were not enriched in our experiments. This may be related to the use of the MLA instead of natural tri-methylated lysine, which can affect binding affinity. Indeed, although recombinant SGF29 specifically interacts with H3K4me3 [32], [33], this protein does not bind to H3K_C_4me3 peptides (data not shown). Interestingly, an uncharacterized protein (KIAA0240) was found to interact specifically with the H3K_C_4me3 nucleosomes. This protein does not carry an annotated putative H3K4me3 interaction domain, indicating that it may interact with one of the H3K_C_4me3 readers. In summary, these experiments reveal that a single histone modification (H3K4me3) contributes significantly to the overall affinity of TFIID for nucleosomes, despite the high basal affinity of the TBP subunit for DNA [34].

### TFIID binding to nucleosomes is enhanced by acetylation of K9/K14 and a TATA box and not disrupted by the presence of H3K27me3

**Figure 2:**
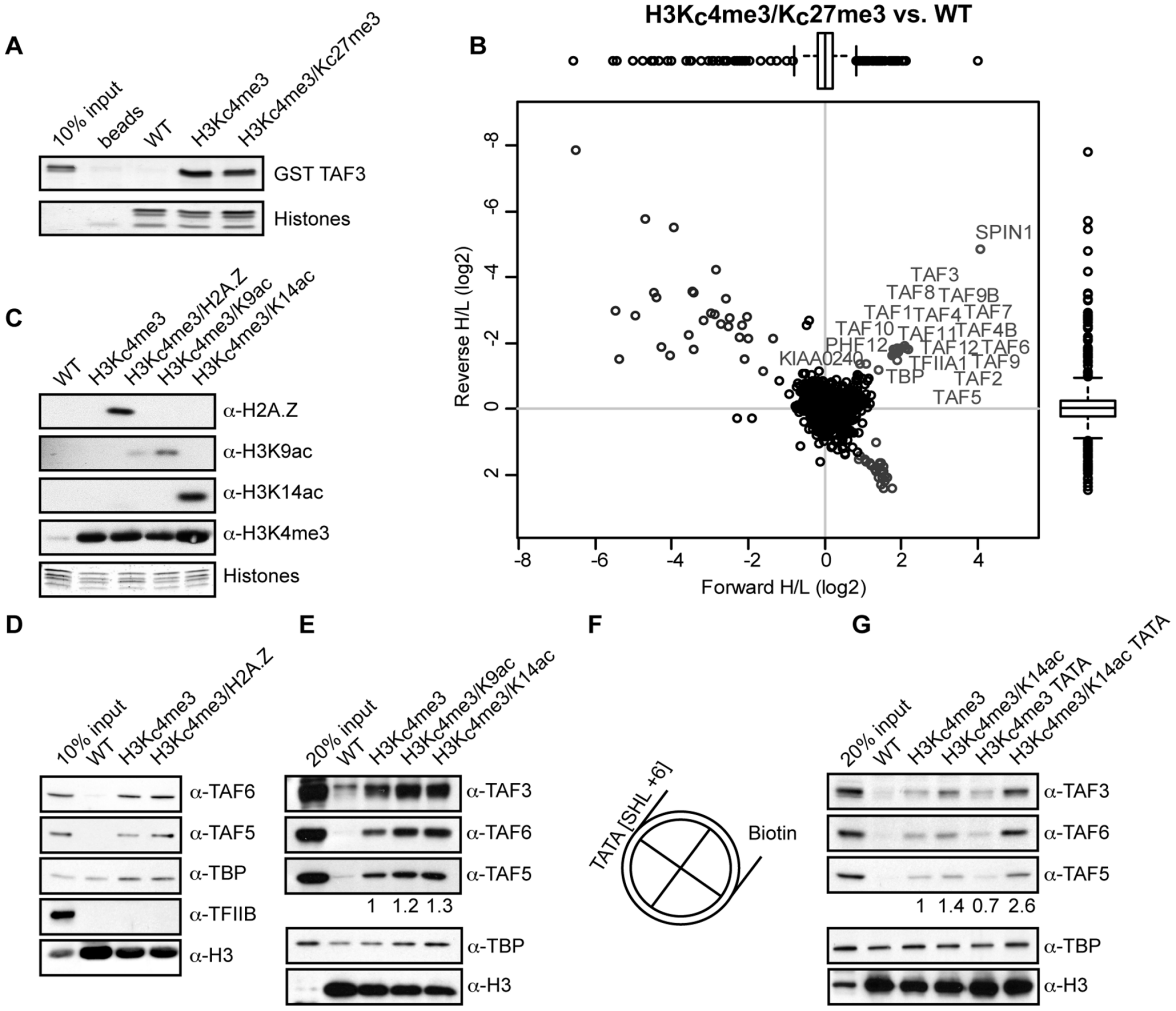
Presence of H2AZ, H3K9/K14ac and a TATA sequence enhances binding of endogenous TFIID to recombinant nucleosomes. (A) Analysis of pulldowns with recombinant nucleosomes immobilized on magnetic streptavidin coated beads and GST-TAF3 (PHD). Proteins are visualized by silver stain. (B) Scatter plot of SILAC ratios for H3K4_C_me3/K27_C_me3 versus non-modified nucleosome interacting proteins. In the upper right corner significant outliers are depicted and labeled in grey based on box plots analysis. (C) Immunoblot analysis of recombinant nucleosomes with the indicated antibodies showing the presence of modifications or variants. (D) TFIID binds to H3K4_C_me3 nucleosomes independently of H2A.Z. Immunoblot analysis of eluted proteins using indicated antibodies. (E) TFIID binding is enhanced by histone H3 acetylation. Immunoblot analysis of eluted proteins using indicated antibodies. TAF5 antibody signal is quantified relative to the H3K_C_4me3 pulldown. (F) Schematic representation of the nucleosome with the introduced TATA sequence and biotin group indicated. (G) Combination of TATA DNA and H3K14 acetylation on H3K4_C_me3 nucleosomes increases the interaction with TFIID. Immunoblot analysis of eluted proteins using indicated antibodies. TAF5 antibody signal is quantified relative to the H3K_C_4me3 pulldown.

The MLA approach can be used to study crosstalk between different chromatin modifications. One such cross-talk phenomenon has been described for embryonic stem cells, where H3K4me3 and H3K27me3 co-occur on silent but ‘poised’ developmentally regulated, bivalent genes [35], [36]. We used both western blotting and quantitative mass spectrometry to study the interaction between TFIID and bivalent nucleosomes. As shown in Fig. 2A, the TAF3 PHD-finger, which directly binds to H3K4me3, binds equally well to H3K_C_4me3- and H3K_C_4me3/H3K_C_27me3 containing nucleosomes. In agreement with this, the TFIID complex was identified as a specific reader for H3K_C_4me3/H3K_C_27me3 marked nucleosomes, as revealed by quantitative mass spectrometry (Fig. 2B). Together, these results demonstrate that TFIID binding to H3K_C_4me3 is not disrupted by the presence of H3K_C_27me3.

SPIN1 and KIAA0240 were again identified as specific interactors, as was TFIIA. Interestingly, PHF2 does not interact with bivalent nucleosomes but another PHD containing protein, PHF12, does. This protein is part of a complex containing the JARID1A H3K4me demethylase enzyme [32], which was not observed as a specific interactor in our experiments. Notably, experiments using nucleosomes containing only H3K_C_27me3 did not yield significant interactors (data not shown).

The chromatin landscape around active gene promoters is characterized by the presence of several distinct features including the histone variant H2A.Z and acetylated histones H3 and H4 [37]. Additionally, distinct DNA-elements in the promoter region can contribute to PIC assembly. We investigated the contribution of these features to TFIID binding *in vitro* using recombinant nucleosomes (Fig. 2C).

Incorporation of the H2A.Z variant marks promoters and enhancers [38]. We first tested the effect of H2A.Z incorporation on TFIID binding to recombinant nucleosomes carrying H3K_C_4me3 (Fig. 2D). This experiment revealed that the presence of unmodified H2A.Z in nucleosomes does not significantly influence TFIID binding *in vitro*. Next, we used an amber codon suppression approach to express recombinant histone H3 containing either acetylated K9 or K14 in bacteria [39] and combined this with the aforementioned MLA approach. Using these doubly modified nucleosomes, we observed enhanced binding (1.2–1.4 fold) of TFIID to nucleosomes decorated with both H3K_C_4me3 and H3K9ac or H3K14ac as compared to H3K_C_4me3 alone (Fig. 2E, G). We find a comparable level of enhancement using either H3K9ac or H3K14ac combined with H3K_C_4me3. These agonistic binding effects can be explained by the tandem bromodomain of TAF1, which was previously shown to interact with double acetylated histone H4 peptides [40]. Furthermore, TFIID binding to histone H3K4me3 peptides has previously been shown to be enhanced by additional H3K9 and H3K14 acetylation [23]. Unfortunately, efforts to express recombinant H3 bearing both H3K9 and H3K14 acetylation proved to be unsuccessful (data not shown).

We then set out to study the potential interplay between histone modifications and specific DNA-elements in the regulation of TFIID binding to nucleosomes. To this end, recombinant nucleosomes were generated containing the classic ‘Widom’ 601 sequence, which carries a weak TATA sequence (GATATATAC) or a 601 variant carrying a consensus TATA sequence (TATATAAAAT) at super helical loop +6 (SHL +6) (Fig. 2F).

As shown in Fig. 2G, H3K_C_me3-dependent TFIID binding is not potentiated in the presence of a consensus TATA sequence (Fig. 2G). However, when the consensus TATA DNA was used in combination with nucleosomes carrying both H3K_C_4me3 and H3K14ac, binding was significantly enhanced as compared to nucleosomes carrying the weak TATA sequence or the methyl/acetyl combination (Fig. 2G). Together, these data reveal that diverse features including histone modifications and specific DNA-elements affect TFIID binding to nucleosomes. Furthermore, the importance of a functional DNA-element with regard to TFIID binding can be dependent on the modification state of the nucleosomes in *cis*, suggesting context-dependent synergy. TBP binding itself seems not affected in these experiments, which may be explained by the nonspecific binding of TBP to unmodified nucleosomes. In these cases, TBP binding is TFIID independent (Fig. 2D, E and G). In these experiments, no competitor DNA was used, which provides a possible explanation for the observed TBP binding.

### Stoichiometry determination of human endogenous TFIID

**Figure 3:**
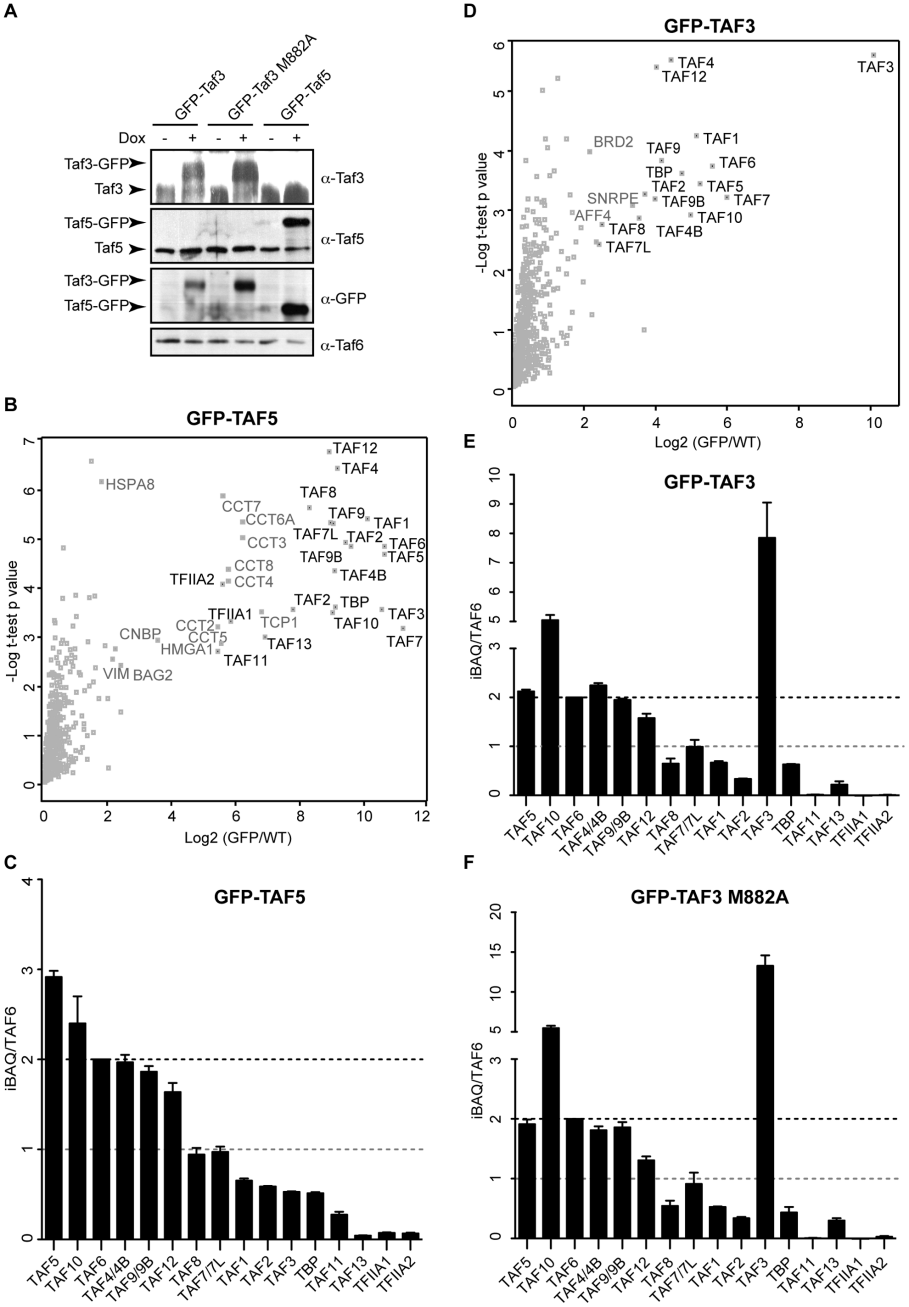
Stoichiometry analysis of endogenous TFIID. (A) Immunoblot analysis of GFP-TAF3, GFP-TAF3 M882A and GFP-TAF5 expression after 24 hours of doxycycline induction with the indicated antibodies (right). Endogenous proteins and GFP-fusions are indicated on the left. (B) Identification of interacting proteins for GFP-TAF5 by volcano plot. The ratio of identified proteins in all fusion lines over wildtype in label-free quantification are plotted against the -log10 of the false discovery rate (FDR) calculated by a permutation-based FDR adapted t-test. In all experiments FDR was set to <0.05 and S_0_ = 1.5. Significant outliers are labeled. (C) Bar graphs indicate the stoichiometry of TFIID subunits (indicated at bottom) relative to TAF6. Black dashed line indicates a ratio to the total TAF6 protein. Error bars indicate the standard deviations of the technical triplicate. (D) Identification of interacting proteins for GFP-TAF3 by volcano plot. (E) Bar graphs indicate the stoichiometry of TFIID subunits (indicated at bottom) relative to TAF6 in GFP-TAF3 (E) and GFP-TAF3 M882A (F) purifications.

So far, we have shown that TFIID is recruited to immobilized nucleosomes containing histone modifications and a consensus TATA box in a synergistic manner. This implies that TAF1 and TAF3, which are the subunits binding acetylated and methylated lysines, as well as TBP, which binds TATA containing DNA, have to be present together in substantial amounts in the same complex. To determine the composition of endogenous human TFIID, we analyzed the stoichiometry using label-free interaction proteomics combined with the iBAQ algorithm, which can be used to estimate relative protein abundance in a sample of interest [41], [42]. We generated a cell line containing a doxycycline-inducible GFP-fusion of TAF5. As a control, wildtype HeLa FRT cells were used (Fig. 3A). Nuclear extracts were made and these were subjected to single-step GFP-affinity purification in triplicate which was followed by on-bead trypsin digestion and LC-MS/MS analysis [43].

Purification of GFP-TAF5 resulted in the identification of all known TFIID subunits (Fig. 3B). iBAQ-based stoichiometry determination revealed the presence of a core complex containing a dimeric TAF6,4,9,10 and -12 module (Fig. 3C). TAF5 appears to be trimeric, which may be due to the moderate ∼5 fold over-expression of the protein (Fig. 3A). Two TAFs, TAF8 and TAF7, are monomeric. The remaining TFIID subunits are substoichiometric, including TAF1, TAF3 and TBP (stoichiometry relative to TAF6 ∼0.5). TAF11 and TAF13 are highly substoichiometric (∼0.2 and 0.05, respectively). Thus, as expected, TAF1, TAF3 and TBP co-purify with core TFIID, although their stoichiometry is slightly lower compared to the dimeric core. This may hint towards the existence of distinct TFIID subcomplexes, each containing a specific set of peripheral TAFs. Alternatively, peripheral subunits may be partially dissociated from the core complex during affinity purification.

To further investigate this, we tagged and purified a peripheral TFIID subunit, TAF3. We also generated a stable cell line expressing a GFP-tagged inducible TAF3 mutant, M288A, which cannot bind to H3K4me3. As was shown for TAF5, purification of GFP-TAF3 and GFP-TAF3 M882A resulted in the co-purification of all TFIID subunits (Fig. 3D,E and F). The stoichiometry determination for wildtype and mutant TAF3 look almost identical, which illustrates the quality of the affinity purifications. Moreover, these data demonstrates that a mutated PHD-finger is not affecting the integrity of the TFIID complex. In both purifications, the stoichiometry of TAF3 is significantly higher compared to the other TAFs, indicating that a proportion of TAF3 is not incorporated into TFIID. Nevertheless, we again identify a stable dimeric core. In addition, the stoichiometry of TAF10 exceeds the dimeric core, which implies the existence of a ‘free’ TAF3/TAF10 dimer. This is in agreement with the fact that TAF10 can associate with TAF3 as well as TAF8 through their respective histone fold domains [44], [45]. Notably, relatively high amounts of TAF1 and TBP co-purified with GFP-TAF3 and GFP-TAF3 M882A (stoichiometry ∼0.5 relative to the dimeric core) (Fig. 3E, F). This indicates that the lysine methyl-, acetyl- and TATA-binding moieties co-exist within a single TFIID complex. These observations therefore substantiate our earlier results in which these three activities were found to act agonistically to anchor TFIID on ‘active’ promoter nucleosomes.

### TAF3 requires its PHD-finger for binding to H3K4me3 in vivo

**Figure 4:**
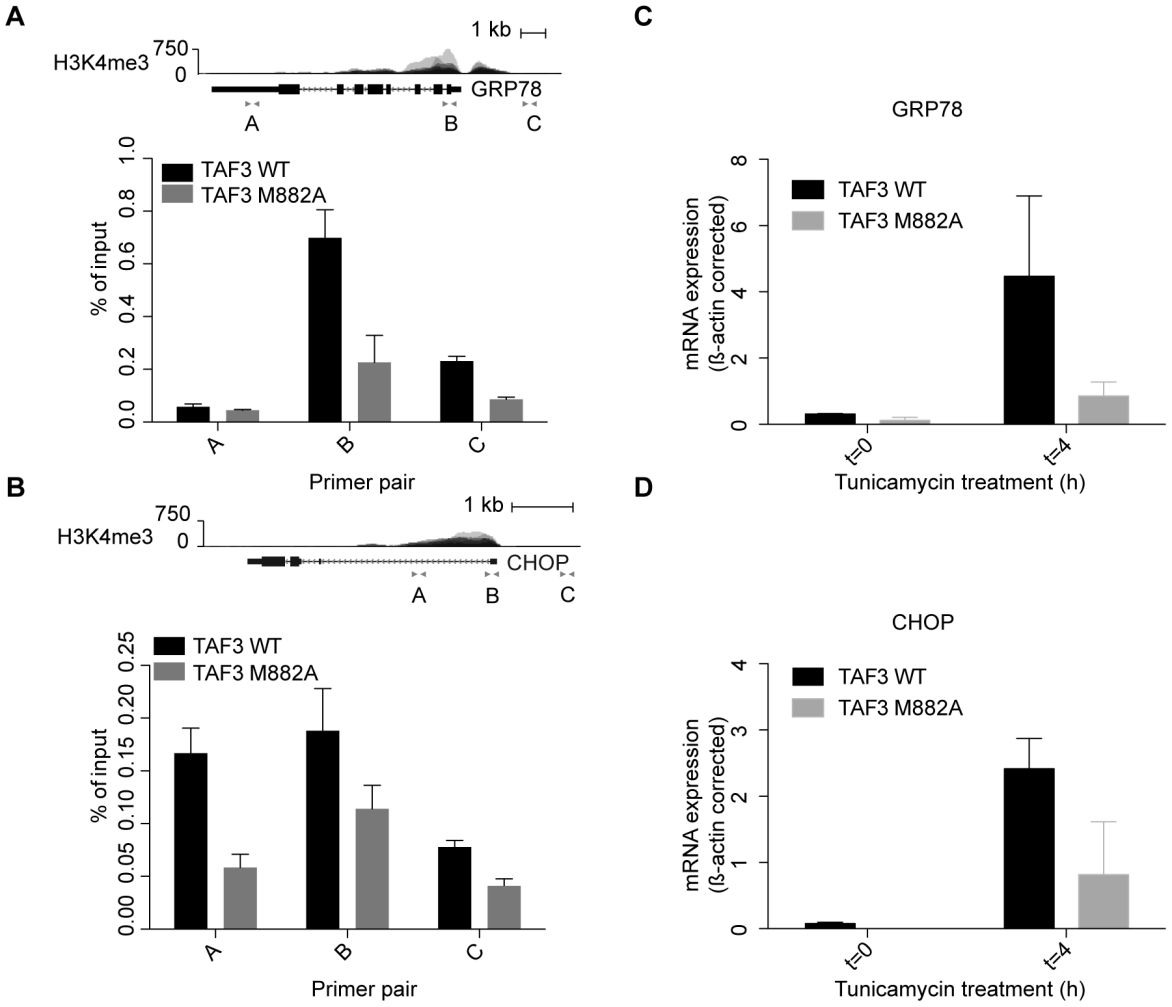
TAF3 binding to ER stress gene promoters is dependent on the PHD-finger. (A) Overlay of ENCODE H3K4me3 profiles from seven human cell lines at the GRP78 locus (upper panel). ChIP analysis of GFP-TAF3 and GFP-TAF3 M882A at GRP78 with the indicated primer sets (lower panel). Standard deviations represent technical triplicates and similar results were observed in at least three independent experiments. (B) Similar labeling as in (A) but for the CHOP locus. (C and D) Analysis of mRNA expression levels of *GRP78* and *CHOP* by quantitative RT-PCR. Levels were normalized to β*-ACTIN* and are presented as change compared to a control DMSO-treated sample. Samples were analyzed 4 hours after tunicamycin treatment. Standard deviations represent two biological duplicates.

We have demonstrated that incorporation of TAF3 lacking a functional PHD domain does not change the composition and stoichiometry of TFIID. Next, we wanted to determine how TFIID recruitment to target sites is affected by the absence of a functional PHD-finger. Recent experiments in our lab have shown that H3K4me3 is present on ER stress responsive genes prior to stress, presumably to maintain these genes in a ‘poised’ chromatin state. Furthermore, in the absence of TAF3, activation of ER stress genes such as *GRP78* and *CHOP* is impaired [46]. Therefore, we used the GFP-TAF3 and GFP-TAF3 M882A cell lines to determine if the PHD-H3K4me3 interaction is important for the binding to and expression of these ER stress responsive genes *in vivo*. Data mining published ENCODE ChIP sequencing data for H3K4me3 in different human cell lines revealed that H3K4me3 is found at the TSS of *GRP78* and *CHOP* in the absence of ER stress (Fig. 4A and B, upper panels). Scanning GFP ChIPs of the *GRP78* and *CHOP* loci revealed that TAF3 binding correlates well with the presence of H3K4me3 (Fig. 4A and B). As was observed for H3K4me3, TAF3 is present at these promoters prior to ER stress, which is indicative of a ‘poised’ state. Strikingly, the TAF3 mutant M882A, which can no longer bind to H3K4me3, shows impaired binding to the ER stress gene promoters (Fig. 4A and B). These results mirror recent findings showing that TAF3 is recruited to a specific subset of promoters enriched for DNA damage response related genes [25].

Next, the inducible GFP-TAF3 and GFP-TAF3 M882A cell lines were used to investigate the mRNA expression of ER stress target genes using the ER stress inducing agent tunicamycin. Strikingly, the induction of both *GRP78* and *CHOP* is impaired in the TAF3 mutant cell line compared to the TAF3 wildtype (Fig. 4C and D). These results indicate that the interaction with H3K4me3 is required for the recruitment to and/or stabilization of TAF3 on ER stress gene promoters *in vivo*. Furthermore, this interaction is required for the rapid induction of ER stress responsive genes. Taken together, these *in vivo* observations strengthen our biochemical data and reveal that interactions with active histone modifications are relevant for TFIID binding to nucleosomes.

## Discussion

The TFIID complex is important for the transcription initiation process and plays a major role in setting up the PIC at pol II promoters. Here, we have shown that synergistic effects of functional DNA-elements and histone modifications mediate high affinity binding of TFIID to promoters.

Stoichiometry analyses of affinity purified TFIID complexes through a core (TAF5) and a peripheral (TAF3) subunit revealed the presence of a stable core complex consisting of two copies of TAF4, TAF5, TAF6, TAF9, TAF10 and TAF12. These results are in agreement with recent work by Berger and colleagues who used recombinantly expressed TFIID (sub)complexes for structural (cryo-EM) studies [7]. Outside of this stable symmetric core, peripheral TAFs appear to be present in substoichiometric amounts. These observations could be indicative of heterogeneity within holo-TFIID complexes, as was suggested previously by Tora and colleagues [47]. Each of these distinct holo-TFIID complexes, all bearing a subset of peripheral TAF proteins, might serve its own specific target genes in a particular tissue or cell state. Indeed, individual TAFs and TAF variants have been shown to specify certain cell fates during development. Deletion of TAF7l in mice, for example, results in defective spermatogenesis [48]. It was also suggested that TFIID adopts different structural configurations dependent on the subunit composition as incorporation of TAF4b induces a more open configuration compared to TAF4 containing complexes [49].

We observed a functional interplay between DNA-elements and histone modifications on TFIID binding to promoter nucleosomes. Interestingly, the positive effect of a canonical TATA box was only apparent in the context of H3K_C_4me3 and H3K14 acetylation. This result could indicate that acetylation of histone tails affects accessibility of the TATA sequence for TBP binding. However, recent structural studies on TFIID binding to a TATA-containing template showed that a structural rearrangement of TAF1/2 in TFIID can be induced by TATA DNA binding [9]. These observations combined with our data suggest that structural changes in TFIID induced by interactions with DNA-elements or histone modifications could result in the exposure of additional chromatin binding surfaces, which would potentiate the complex for multivalent engagement. Additionally, the spacing between the +1 nucleosome and the TATA element could affect TFIID binding to different promoters in a specific manner [12].

We used ChIP experiments to show that TAF3 binds to the promoters of ‘poised’ stress genes. This binding is severely compromised when expressing a TAF3 mutant containing a point mutation (M882A), which cannot bind to H3K4me3. Roeder and colleagues recently showed that knock-down of TAF3 only results in a minor change in global pol II-dependent transcription. However, for the induction of early p53 response genes as well as ER stress genes, the interaction between TAF3 and H3K4me3 appears to be critical [25]. Together these data illustrate that H3K4me3 binding by TFIID is only required when rapid induction of transcription is demanded. When unchallenged, acetyl and TATA binding can be sufficient for TFIID loading. These experiments from Roeder and colleagues, together with our data, further suggest that H3K4me3 can act either independently or cooperatively with a TATA box to regulate PIC formation and transcription. This, together with the fact that only ∼10% of human pol II promoters contain a canonical TATA box, implies a certain degree of structural plasticity regarding TFIID engagement on different target genes. Interestingly, yeast TFIID lacks an H3K4me3 binding domain. Nevertheless, recent high resolution ChIP in yeast revealed that TFIID binding partially overlaps with the position of the +1 nucleosome [12], indicating that TFIID can bind simultaneous to the nucleosome depleted region and to the first nucleosome. This observation is more pronounced on Taf1 depleted genes, indicating that SAGA and TFIID regulated genes are different in promoter architecture, at least in yeast [50]. A systematic analysis of human promoter architecture and TFIID association however remains to be done.

Future experiments can be directed towards determining the exact position of TFIID subunits in promoter bound complexes. Furthermore, functional domain mapping experiments could be pursued to dissect the molecular mechanisms underlying the multivalent engagement of TFIID at various promoter nucleosomes. Additional stoichiometric analysis on nucleosome bound TFIID using different promoter and/or enhancer nucleosomes for affinity purification could reveal the exact composition of the TFIID complex binding to these nucleosomes. In these experiments, we only made use of ‘601’-based DNA sequences to avoid nucleosome sliding and transcription factor binding. Future experiments using promoter DNA sequences should reveal the contribution of additional DNA-elements to TFIID binding. Finally, deciphering the genome-wide profile of individual core and peripheral TFIID subunits in different cellular systems and stress conditions will increase our understanding regarding the assembly and composition of TFIID (sub)complexes and their role in the regulation of transcription initiation.

## Materials and Methods

### Plasmids and cell culture

The ORF of the bait protein was amplified by PCR using the relevant human cDNA constructs and introduced into pDONR2.1. The DNA sequence of the amplified ORF was verified and introduced into a GATEWAY-compatible version of pCDNA5/FRT/TO essentially as described before [51]. Mouse TAF3 and mutant M882A were tagged by GFP at the N-terminus. Stable doxycycline-inducible cell lines were created by transfecting pCDNA5/FRT/TO and pOG44 into HeLa FRT cells carrying the TET repressor using polyethyleneimine followed by antibiotic selection. Cells were grown in DMEM with high glucose supplemented with pen/strep and L-Glutamine (all LONZA) under blasticidin and hygromycin B selection. pRPN-mTAF3 (PHD) was described previously [23]. pDUET-H3K4C_,_/K14X was derived by introducing K4C, C110A mutations and an amber codon at position 14, into Drosophila melanogaster histone H3. All histone H3 plasmids carried a C110A mutation. Amber codon histones were inserted into the pDUET plasmid using Nco1/Xho1 and transformed in bacteria that already carried pAC-KRS (kind gift of Robert Schneider). Point mutations in H3 were introduced using the Quickchange protocol (Stratagene) and verified by DNA sequencing. Other histone proteins were expressed from pET21b (gift from Y. Bai).

### GFP affinity purification and sample preparation

Extract preparation [52] and affinity purifications using GFP-beads [43] were performed essentially as described before. Briefly, nuclei were isolated using hypotonic lysis and nuclear extracts were prepared by using 420 mM NaCl Purifications for GFP lines and WT HeLa cells were performed in triplicate using 1 mg of nuclear extract per purification and GFP binder beads (CHROMOTEK) in 20 mM HEPES-KOH pH 7.9, 20% glycerol, 300 mM NaCl, 2 mM MgCl_2_, 0.2 mM EDTA, 0.1% NP-40, 0.5 mM DTT and complete protease inhibitors (Roche). All purifications included 50 μg/ml ethidium bromide to suppress DNA mediated interactions. After 2.5 hours incubation at 4°C the beads were extensively washed and on-bead digestion was performed using 0.1 μg trypsin (Promega).

### Protein expression and nucleosome reconstitution

GST-mTAF3 PHD was expressed in E. coli strain BL21DE3 at 37°C in LB medium. Drosophila histones were expressed in E. coli strain BL21DE3 codon+ or Rosetta and prepared essentially as previously described [53]. For expression of acetylated histones 20 mM Nicotinamide and 10 mM N-acetyl-L-Lysine (Sigma) was added to the cultures at OD_600_ = 0.6 and protein expression was induced after 30 minutes using 0.5 mM IPTG as described before [39]. Histone H3K4C and derivatives were alkylated essentially as described before [29], [54]. 167-bp DNA (‘601 Widom’ positioning sequence or TATA mutants) was produced by PCR amplification using one biotinylated primer, purification using DEAE anion exchange and ethanol precipitation. After octamer refolding, nucleosomes were reconstituted with the DNA using salt displacement.

### Extract preparation and nucleosome pulldowns

Hela S3 cells were cultured in large quantities using a bioreactor setup in MEM depleted from Lysine and Arginine supplemented with dialyzed FBS, Pen/Strep, L-Glutamine (all LONZA) and normal or ^13^C^15^N- labeled arginine and lysine (Isotec). Nuclear extracts were prepared by isolating the nuclei and hypertonic lysis as described before [23], [52]. For nucleosome pulldown assays magnetic Streptavidin beads (Sigma, MyOne) were coated with 130 pmol nucleosome and incubated in pulldown buffer (20 mM HEPES pH 7.5, 150 mM NaCl, 0.2 mM EDTA, 20% glycerol, 0.1% NP-40 and 1mM DTT) for one hour at 4°C. After washing twice, 500 µg nuclear extract was added and beads were incubated for 2–3 hours rotating at 4°C. For mass-spec experiments, heavy-labeled modified nucleosome pulldowns and light controls were mixed at this point. Proteins were eluted from the beads in sample buffer after extensive washing and the bound proteins were analyzed by immunoblotting or processed for LC-MS/MS. Peptide pulldown experiments were performed essentially as described [23]. Briefly, biotinylated peptides were alkylated as described before [29] and incubated with magnetic Streptavidin beads (Sigma, MyOne). After incubation and extensive washing beads were incubated with GST-TAF3 (PHD) lysate. Bound protein was visualized using Coomassie blue staining.

### Mass-spectroscopy

Eluted proteins were separated on a SDS-PAGE gel and stained using Colloidal blue staining (Invitrogen). Lanes were sliced into eight pieces and samples were subjected to in-gel digestion with 0.1 µg trypsin (Promega) as described before [23]. Tryptic peptides were extracted from the individual gel slices and concentrated using stage-tips with C18 material. The peptides were applied to online nanoLC-MS/MS, using a 120 minutes acetonitrile gradient. Mass spectra were recorded on a LTQ-Orbitrap-Velos mass spectrometer (Thermo) selecting the 15 most intense precursor ions of every full scan for fragmentation. The data was analyzed using the Max-Quant software package [55].

### Chromatin immunoprecipitation

Cells were cross-linked at 80–90% confluency using 1% paraformaldehyde in PBS for 10 minutes at room temperature. Reactions were quenched by addition of 125 mM glycine for 5 minutes on ice. After a cold PBS wash cells were scraped and collected by centrifugation (5 min, 400 g, 4°C). Pelleted cells were resuspended in ChIP lysis buffer (1% SDS, 10 mM EDTA, 50 mM Tris-HCl pH 7.9, 1 mM DTT, 5 µM sodium butyrate (Merck) and complete protease inhibitors (Roche)) and disrupted by sonication (Bioruptor, Diagenode: seven cycles, 30 seconds on/off, high setting) to produce an average DNA fragment size of ∼400-bp. Samples were centrifuged (5 minutes, 200 g, 4°C) and supernatant collected. For immunoprecipitation, chromatin was diluted in IP buffer (0.5% Triton X-100, 2 mM EDTA, 20 mM Tris-HCl pH 7.9, 150 mM NaCl, 1 mM DTT, 5 µM sodium butyrate and complete protease inhibitors (Roche)), 1–5 µg antibody was added and rotated overnight at 4°C. Immunocomplexes were collected for 4 hrs at 4°C on protein A/G PLUS-agarose beads (Santa-Cruz), after o/n blocking in 1.5% fish gelatin and washing. Subsequently beads were washed four times at 4°C with wash buffer (0.25% NP-40, 0.05% SDS, 2 mM EDTA, 20 mM Tris-HCl pH 7.9, 250 mM NaCl, 5 µM sodium butyrate and complete protease inhibitors) and once with TE (10 mM Tris-HCl pH 6.8, 1 mM EDTA). Cross-links of protein-DNA were reversed by overnight incubation at 65°C in 100 µl elution buffer (100 mM NaHCO_3_, 1% SDS). Samples were treated with 1 mg/ml proteinase K (Roche) and 1 mg/ml RNase A for 2 hours at 37°C. DNA was purified using PCR purification kit (Qiagen) and amplified in a 25 µl reaction mixture (iQ SYBR green supermix (Biorad)) in a real-time PCR machine (CFX96, Biorad). Primer sequences are available upon request.

### mRNA expression analysis

Total RNA was isolated using RNeasy kit (Qiagen) and cDNA was synthesized using the First-strand cDNA synthesis kit (Qiagen) both according to the manufacturers manual. Subsequently the cDNA was amplified in a 25 µl reaction mixture (iQ SYBR green supermix (Biorad)) in a real-time PCR machine (CFX96, Biorad). Primer sequences are available upon request.

### Antibodies

α-TBP (SL30) (gift from Henk Stunnenberg), α-TAF3 and α-TAF5 (obtained from Robert Roeder), α-TAF6 (25TA-2G7, Euromedex), α-TFIIB (Santa Cruz), α-H3 (Abcam Ab1791), α-H3K4me3 (home made), α-H3K9ac (Cell Signaling 9671), α-H3K14ac [56] and α-H2A.Z (Abcam Ab18263) were used for immunoblotting. For ChIP an immunoblotting α-GFP (gift from Geert Kops) was used. Quantification of antibody signals was performed using Adobe Photoshop.
